# From a Novice Teacher to a Teacher Leader: An English-As-a-Foreign-Language (EFL) Teacher’s Cognitions About Her Professional Development

**DOI:** 10.3389/fpsyg.2022.921238

**Published:** 2022-06-21

**Authors:** Lori Xingzhen Gao, Jennifer Jie Yang

**Affiliations:** College of Foreign Languages, Taiyuan University of Technology, Taiyuan, China

**Keywords:** language teacher cognition, professional development of English language teachers, in-service EFL teachers, teacher learning, Chinese context

## Abstract

In this paper, a qualitative study was conducted on Jennifer, an EFL teaching professional at the tertiary level in a Chinese context, to investigate her cognitions regarding her professional development and accompanying facilitating factors in her journey from a novice teacher to a teacher leader with Borg’s model of language teacher cognition as the conceptual basis for subsequent analysis. Jennifer’s written overview and verbal narration in interviews of her journey in professional development were gathered following guiding protocols. Collected data were processed with thematic analysis in NVivo 12. Findings suggest that Jennifer has clear cognitions about how she learned and improved, i.e., the positive changes, in her professional journey and the facilitating factors that mediated her improvement and progress. These facilitating factors were found to include her teaching experience, in-service training, administrative promotion, drawing wisdom from reading Chinese classics, and constant reflection upon her English as a foreign language (EFL) teaching, etc. The implications of the present study for language teacher cognition researchers, English language teacher educators, and EFL teachers in the Chinese context were also discussed.

## Introduction

English language education in 21st century China has adopted a top-down approach from policy-making by the Ministry of Education (MoE) to policy implementation at educational institutions at all levels. In 2001, the MoE stipulated that, English classes start in the third grade of primary school. In some larger cities, children have the chance to start learning English through songs, games and toys in kindergarten. In secondary schools, 30% of the teaching time is allocated for language learning: Chinese and one foreign language (English in most cases) (Gil and Adamson, 2011). The syllabus emphasizes students’ development of international perspectives and strengthens patriotism through foreign language learning with selected materials (Wang, 2007). The new millennium has witnessed English developing into one of the most widely taught foreign languages in China. The English language, in addition to its consistent role in modernization, provides “English for international stature” (Lam, 2002, pp. 246–247), as China’s mounting participation and engagement in international affairs calls for high proficiency in foreign languages. International conferences and activities held in China such as Olympic Games Beijing 2008, the World Expo in 2010, Olympic Games Beijing 2022 as well as China’s entry into the World Trade Organization (WTO), has increased enthusiasm for learning English among Chinese people who want to communicate with foreign visitors. English is currently enjoying a status unsurpassed in the Chinese context, and English Language Teaching (ELT) is reaching increasing prominence at all levels in the education system in China.

The rapidly increasing population of students and people yearning for English skills, development of a national English curriculum, and ongoing educational reform throughout China all place increasing demands on qualified English teachers who “require a combination of competencies and backgrounds that may be unprecedented” (Curtain and Pesola, 1994, p. 241). English language teacher education and professional development are increasingly crucial and have become a vital research focus. Richards (1987) observation that “the intent of second language teacher education must be to provide opportunities for the novice to acquire the skills and competencies of effective teachers and to discover the working rules that effective teachers use” (p. 15) sheds light on initial training and ongoing teacher education. As instrumental, English language teachers’ knowledge, belief, thinking, and perception about the factors related to teaching and learning affect their teaching practices. English teachers’ personal knowledge, i.e., the capability to reflect on and analyze their instructional behaviors and insights and values that inform their teaching, in addition to content knowledge (knowledge and skills concerning the subject matter), knowledge of learners, pedagogical knowledge (knowledge concerning principles of teaching and learning), functions as a basis for their continuing professional development.

Based on English language education and English language teacher education in China; this paper investigates the cognitions of Jennifer, a Chinese university English as a foreign language (EFL) teacher, assessing her professional development to unveil secrets about her learning and improvement and interpret the facilitating factors of her improvement and progress in her career. Using this as a case study to examine professional development of in-service English teachers in China thereby providing implications for language teacher cognition researchers, English language teacher educators, and English teachers, enables more effective use of teacher training.

## Literature Review

### Professional Development of English Language Teachers

Teachers as the main medium of classroom instruction play a crucial role in education (Krashen, 1982) subsequently supporting workers in society. Therefore, teachers professional development is instrumental to teaching success and positive development of society. Valuing teachers in the sense of cultural inheritance as well as talent training, governments put enormous investment in teacher training and relevant research, endeavoring to help teachers reach their full potential in classroom instruction. Dos Santos (2016) explored foreign language teachers’ professional development through peer observation. Postholm (2008) described the key function of reflection in teachers’ learning and explored teachers’ professional development from a theoretical perspective. According to Watson and Darasawang (2020), students’ learning outcomes have been expected to be enhanced under the guidance of teachers with updated innovative teaching ideas and approaches. Poonpon (2022) examined English language school teachers’ professional development needs using a Likert-scale questionnaire and found that English teachers need to develop language proficiency in curricular areas, pedagogical skills, and technology-related teaching approaches. Liu et al. (2020) investigated the role of overseas academic visiting in language teacher development. Beginning EFL teachers’ emotional labor strategies (Li and Liu, 2021), EFL teachers professional qualities (Chu et al., 2021; Liu et al., 2021b), and EFL teacher resilience (Liu and Chu, 2022) in a Chinese context were also explored. Ji et al. (2022) explored pre-service teachers’ pedagogical decisions on integrated-skill instruction in a Chinese context.

Publicized research about professional development of English language teachers suggests that the success of English language teaching professionals from novice teachers is the result of a manifold factors. On the part of the schools and universities, these include mentoring novice English language teachers in their initial years of classroom teaching, offering financial support for in-service training and learning, and creating opportunities to pursue advanced degrees, etc. (Darling-Hammond, 2010a; Dillon, 2011). On the part of English language teachers, their English language learning experiences at schools, professional coursework, and classroom teaching experience, contribute to their ability and skills improvement in professional foreign language instruction. In addition, English language teachers’ self-knowledge, i.e., their capability to reflect on and analyze their instructional behaviors and insights and values that inform their teaching, along with content knowledge (knowledge and skills concerning the subject matter), knowledge of learners, pedagogical knowledge (knowledge concerning principles of teaching and learning), also functions as a basis for further professional development.

### Language Teacher Cognition

Scholars have long acknowledged (for example, Sizer, 1966; Shulman, 1992; Darling-Hammond, 2010a) that teaching takes center stage in all educational institutions and teachers are expected to have the competence and potential to make a difference in student learning (Darling-Hammond et al., 2001; Fetler, 2001; Rivkin et al., 2005). Since the development of cognitive psychology, teachers have been regarded as thinkers rather than teaching technicians, and research on teaching and teachers has switched from teachers’ teaching behaviors to their cognitions about teaching. Teachers’ attitudes, knowledge, beliefs, thoughts, and decision-making are now, collectively referred to as “teacher cognition,” especially in the field of second/foreign language education (Pajares, 1992; Calderhead, 1996; Borg, 2009, 2015).

Research on language teacher cognition commonly has been conducted in curricular areas, such as L2 grammar (Sata and Oyanedel, 2019), writing (Ngo, 2018), speaking (Webster, 2019), listening (Gao et al., 2019), translation (Wu et al., 2019, 2021), and pronunciation (Couper, 2019), etc., and at various levels from kindergarten to adult education (e.g., Yan, 2020; Liu and Han, 2022). Language teacher cognition is contextually specific and affected by various factors. Personal factors based on a teachers’ understanding of their own teaching behaviors help define teacher cognition (Clandinin and Connelly, 1987; Sun and Zhang, 2019). In addition, factors, such as “teachers’ personality factors, educational principles and research-based evidence” (Richards and Lockhart, 1996, p. 30), different professional stage (Berliner, 1994; Kang and Cheng, 2014), teachers’ emotions (Zembylas, 2005), and factors such as COVID-19 (e.g., Gao and Zhang, 2020; Wu and Wei, 2021) also have a recognizable effect on teacher cognition. According to Borg (2015), language teacher cognition is affected by three factor types: schooling, professional coursework, and contextual factors. Ozturk and Gurbuz (2017) used a grounded theory approach to re-define language teacher cognition within a broader perspective through a data-driven model with three case studies, putting forward a framework conceptualizing the notion of language teacher cognition, i.e., how language teachers construct their cognition on language teaching. Li (2020) and Zhan and Jiang (2021) explored the diverse and dynamic contexts of language teacher cognition and the relationship between language teachers’ thinking and doing from a sociocultural perspective.

Studies of language teacher cognition mainly focus on conceptual and terminological understandings of teacher cognition (e.g., Shulman, 1986; Borg, 2003; Kubanyiova and Feryok, 2015), factors affecting the development of teacher cognition (e.g., Li and Liu, 2021; Liu et al., 2021a, 2022), relationships between teacher cognition and their actual classroom instructional practices (Borg, 2015; Bao, 2017; Chen, 2017; Sun, 2017). Teachers’ voices, in contrast, remain severely under researched. As such, language teacher cognition research is missing this key stakeholder perspective of who is in a prime position to report “on-the-ground” teachers’ cognitions about their professional development. This research addresses this gap by conducting an in-depth qualitative study which embraces the teacher’s cognitions providing her professional development over the past 28 years offering EFL and language teacher cognition researchers a direct voice. To be more specific, the paper addresses the following research questions:

(1) What are the changes in the EFL teacher’s cognitions about her professional development?
(2) What are the factors that mediated these changes, and how did the factors mediate the changes?

## Materials and Methods

### Participant and Research Context

The teacher (Jennifer) in the present study started her career as an EFL teacher in a university in northern China in 1994 and is now the director of the Department of Foreign Languages. As Jennifer’s former student in university from 1998 to 2002 and now her colleague, the researcher (the first author) was able to witness Jennifer’s professional development over the past 24 years, establishing objective verification to the narration of her written overview and interviews regarding her experience with professional development, adding reliability to the present study. Jennifer’s narration provides data about how she developed from a novice teacher to a teacher leader where she could reflect on the teaching and learning of English as a foreign language (EFL) to her students.

Jennifer graduated from a provincial university in 1994 and since then has been working as an EFL teacher in the Department of Foreign Languages in a university in northern China. She divided her professional career into three consecutive stages. In the first stage, she was a novice teacher; in the second stage, she taught in her “comfort zone” as an experienced teacher; and in the third stage, she was promoted to director of the Department while working as a well-developed EFL teacher. In the first two stages She taught such courses as *Integrated English, Advanced EFL Listening, Basic/Intermediate/Advanced EFL Writing, and Introduction to Chinese Culture*, etc. to freshmen and sophomore English majors. While in the third stage she taught *Chinese Language and Culture* to Masters of Translation and Interpretation (MTI) students and she also taught Integrated English to English majors. The Department of Foreign Languages where Jennifer works possesses a teaching and learning environment which can be described as serious-minded, enquiry-oriented, and learning- centered.

### Interviewing as a Research Method

As a tool for qualitative data collection for applied linguistic research, interviews deliver in-depth exploration into the topic at hand (Miles et al., 2014). Dörnyei (2007) states that with the degree of structure as the classification standard, interviews can be grouped into structured, unstructured, and semi-structured interviews. Semi-structured interviews, based on compromise between the two extremes, provide pre-prepared guiding questions with an open-ended format where the interviewee is encouraged to give a detailed account of the issue raised. Creswell (2014) states, qualitative interviews can be conducted face-to-face with the interviewee, over the telephone, in focus groups, or via Internet such as emails. In qualitative interviews, the interviewee can provide historical information and the interviewer controls the line of questioning.

Interviews are suitable for the present study because its purpose was to explore an EFL teacher’s cognitions about her professional development and the factors facilitating her teaching career. The semi-structured one-to-one face-to-face interviews were adopted as the inquiry instrument. The first author was the interviewer who used two broad and open-ended questions to guide the consecutive interviews: (1) How do you reflect on the changes in your professional development? (2) In your cognition, what are the factors that have facilitated the changes? And how? Jennifer, the participant, was encouraged to elaborate in a detailed way on her reflections regarding the two questions. The interviewing instrument as Zhang (2010) mentioned, is a naturally and socially acceptable way to gather in-depth data on issues in various situations.

### Roles of the Researchers

In the present study, the first author, also Jennifer’s former student and present colleague, has experience in performing academic research, took the role of lead researcher designing and governing the data collection, writing up the first draft and subsequent revisions. Moreover, as previously mentioned, the first author witnessed Jennifer’s development as a novice teacher, and in a colleague’s position, Jennifer’s growth as an experienced EFL teacher as well as the director of the Department. Jennifer, an EFL teaching expert, who reads Chinese classics, spares little of her time and effort focused on academic research. In the present research she was encouraged to reflect on her professional development since she started teaching EFL teacher 28 years ago and to recount her experience in a chronological order in both a written description and subsequent interviews. Communications between the authors were carried out in Chinese since Chinese as their first language served the best function for communication.

### Data Collection and Analysis

The present study collected qualitative data from multiple sources including the participant’s written experiential overview, in-depth semi-structured one-to-one face-to-face interviews, the participant’s teaching plans, and certificates. Multiple data sources were chosen because they made triangulation possible. Triangulation is “a qualitative research strategy to test validity through the convergence of information from different sources” (Carter et al., 2014). Furthermore, it is seen by researchers as more than a research tool: It is also a solution to obtain valid and reliable data. Data collected centered on Jennifer’s life stories of her professional development are all in Chinese. Jennifer’s narratives provide insight into her introspective cognition of her professional development related to EFL teaching.

Data collection was conducted in two major phases, occurring over half a year from June 2021 to March 2022. In the first phase, from June to November 2021, Jennifer reflected on her past teaching experience and produced a written overview of over 5,000 Chinese characters about her professional life since her first day as an EFL teacher in a university according to her self-prescribed chronological structure and terms that emerged in her process of meaning-generalization of her work life. Data from Jennifer’s written reflection were processed preliminarily. In the second phase of data collection, from December 2021 to March 2022, based on themes that had been identified from the preliminary data analysis of her written overview, Jennifer was interviewed three times to elicit verbal data centering on changes in her professional development and facilitating factors. Each interview last from 50 min to 1 h.

Data analysis procedures started immediately after the written reflection was read. Audio data from interviews were transcribed and processed, and field notes taken during interviews were clarified and summarized. Thematic analysis, “a method for identifying, analyzing and reporting patterns” (Braun and Clarke, 2006; Miles et al., 2014; Gao and Zhang, 2020, p. 6), was used to scrutinize data for typical themes and concepts (Miles and Huberman, 1994; Rubin and Rubin, 2011). During the data cleansing process, Chinese verbal data obtained from written reflection and interviews were not translated into English because Chinese, as the first language of Jennifer and the authors, can clearly convey their ideas and insights. If data translation is done before processing, some information may be lost as a result of the lack of equivalent vocabulary, syntax and concepts between Chinese and English (Sechrest et al., 1972). In order for the findings to be understood by international readers, all data were presented in English. NVivo 12 was utilized during data analysis for coding, and generating themes. First, the interview transcripts were imported into NVivo. Second, textual data were read several times for coding purposes. Hundreds of codes were generated, including “using dictionaries for lesson preparation,” “referring to the teacher’s book,” “an analogy to calligraphy,” etc. Third, themes were abstracted from these codes, such as “lesson preparation,” “cognitions about EFL teaching,” etc. Fourth, the themes were then classified into categories following the participant’s defined professional development stages. The utilization of NVivo 12 was of substantial assistance as the software helped visualize the data analysis process and outcome and demonstrated the relationship among the categories of themes.

## Findings: Jennifer’s Cognitions About Her Professional Development

During the repeated reading and coding of Jennifer’s written overview and interviews about her professional development from her days as a novice EFL teacher to a teacher leader, the following themes emerged by themselves as Jennifer’s cognitions about EFL teaching, her role(s) in teaching EFL, and her relationship with students. These themes manifested as Jennifer unfolded her experience as an EFL teacher. The following sections report Jennifer’s cognitions about her professional development over the past 28 years, during which three stages were identified by Jennifer: The first stage as a novice teacher (1994–2002), the second stage as an experienced teacher (2003–2009), and the third stage as a teacher leader – the (associate then principal) director of the Department of Foreign Languages (2010- present).

### Jennifer’s Cognitions About Her Professional Development in the First Stage

As reflected in the written overview and interviews, in her early years as a novice EFL teacher, Jennifer felt she lacked confidence in several aspects of her professionalism, including her cognition about EFL teaching, her role in EFL teaching, and her relationship with students. The words and/or phrases in Chinese such as “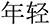 (young and inexperienced),” “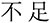 (inadequacy),” “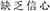 (the lack of confidence),” “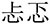 (perturbation),” “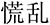 (flurry and bewilderment),” and the like, appeared frequently.

#### Jennifer’s Cognitions About the First Stage of English as a Foreign Language Teaching

According to Jennifer’s verbal data from interviews, when she started as an EFL teacher of college English for non-English majors in 1994 after graduating from a provincial university at the age of 23, she considered herself an incompetent EFL teacher lacking confidence as a result of perceived inadequacy in English proficiency. She believed that objectives of EFL teaching should be explaining texts clearly in terms of vocabulary and grammar and being recognized by students; and that a teacher who could realize these objectives would be a qualified teacher. She was frustrated to find that there was a gap between knowledge learned in university and her EFL teaching practice in the classroom. She tried to bridge the gap through personal endeavor. What follows is her recounting of her insight and feelings of her inadequacy in this stage:

*In the beginning days of my teaching career, there was severe inadequacy in terms of knowledge reserve, teaching experience, and psychological preparation. I was perturbed every time I stood in the front of the classroom, facing the students who were about my age. I was very happy when I explained the texts clearly and obtained recognition from students. Being a teacher was like a mountain whose true features were starting to be revealed little by little as I climbed the stairs.*

Jennifer repeatedly mentioned her feelings of anxiety and worry when she was stuck by questions from students for which she did not know the answer. With a Bachelor’s Degree of Arts, Jennifer was not adequately equipped with pedagogical content knowledge and recognized her EFL teaching as struggling and frustrating. Spurred on by her cognitions about her incompetence and inadequacy, Jennifer was concerned for the implementation of lessons. She spent substantial amounts of time preparing teaching materials. She mentioned that an English-Chinese dictionary was the main tool for lesson preparation:

*The words in the texts were sometimes confusing. I found myself unable to fully understand the literal meaning of words in the texts even with the teachers’ book. I frequently turned to the great dictionary (referring to her English–Chinese dictionary) when preparing lessons. I was focusing on making out the literal meaning of words in the texts.*

In Chinese folk wisdom, there are many two-part allegorical sayings. One of these is “dumplings boiled in the teapot – difficult to be poured out,” indicating someone who knows a lot but is inarticulate in expressing their knowledge to his or her audience, which suits the situation of a novice teacher like Jennifer. She also made an analogy between her EFL teaching in the initial stage to making dumplings.

*Dumplings boiled in a teapot cannot be poured out easily; to avoid this dilemma, I attempted to “boil dumplings in a saucepan.” However, my problem is that I didn’t have proper “dumplings” for the lack of necessary meat and vegetables.*

By “the lack of necessary meat and vegetables,” Jennifer means she had a tough time teaching as a novice teacher because she did not have the necessary knowledge reserve. This inadequacy in English proficiency was accompanied by an un-suppressed joy when she acquired new knowledge in her autonomous learning of the texts with dictionaries and *The Teacher’s Book* (which matched *The Students’ Book* as teaching aid) as main knowledge and information sources.

#### Jennifer’s Cognitions About Her Role(s) in English as a Foreign Language Teaching in the First Stage

Guided by her cognition about the major objective of EFL teaching in this stage, Jennifer positioned herself as a “lecturing teacher” and a “teacher-learner” and buried herself in texts. She described herself as “hunted” and pushed by her anxiety about the subject matter and the students’ learning needs instead of as a “hunter” motivated by her own inner desire and passion for EFL teaching. What she did with the texts was focusing on creating meanings out of words without going any deeper into them. She made an analogy between EFL teaching and a trip in which she found many new things, marveling at them but having no time or competence for comprehension. She could not find the focus of the texts she was teaching. This led to her second role as a “teacher-learner” during which she audited experienced teachers’ classroom, assisting her in her improvement in classroom instructions:

*With constant accumulation of learning from experienced teachers, I gradually updated teaching skills and became clearer about the focus of texts. I made progress in classroom teaching and become more confident.*

In addition to regular college English classes, Jennifer was also assigned by the Department to teach the College English Test Band Four (CET-4) and College English Test Band Six (CET-6) classes for non-English majors. She was not confident in her English proficiency at the beginning.

*I did not attend CET-4 and CET-6 myself; as a matter of fact, I was uncertain whether I could get high marks in these tests if I had the chance to try. I summoned up my courage and accepted the teaching task as the Department director assigned it to me.*

Driven by her uncertainty and desire to improve her mastery of CET-4 and CET-6 knowledge and test-taking skills, she spent time studying past CET-4 and CET-6 exams before she started the classes. She found CET-6 passages were not easy, which motivated her to learn and read more to prepare her for EFL teaching. She was motivated to learn based on her cognitions of her lack in English proficiency and test-taking skills.

#### Jennifer’s Cognitions About Her Relationship With Students in the First Stage

Jennifer’s cognitions about her relationship with her students as a novice teacher contain two complementary components. On one hand, she regarded herself as accepted by her students due to the small age difference between them. In Jennifer’s eyes, that acceptance was based on nothing but her young ego.

*I received acceptance from the students during my perturbation. They appreciated the enthusiasm and flexibility in my young ego.*

This acceptance based on small age differences instead of admiration for her knowledge and teaching competence (which she lacked in this stage) sometimes made her students go too far in approaching her, which to her was barely acceptable. Jennifer recounted one experience that made her feel startled and annoyed with her students in that stage:

*I was once followed by the students to my mother’s house. One Sunday a group of students poured in for a visit. When I asked them how they knew my address, they laughed my question off. A casual conversation later on with the kiosk keeper near the gate revealed that two young men asked him about my address and he told them because he thought they were potential guests for my wedding. It turned out that the two guys were sent there by their peers to find out my exact address.*

Jennifer believed that one of the reasons why her students kept so close to her was the small age difference. In 1990s, the admission rate of college entrance examination across China was around 35%, and some students were admitted as college students after several attempts at the college entrance examination, which means they spent extra time in high school. Those students were a few years older than their peers in college classes and even as old as or older than teachers fresh from universities like Jennifer. They treated Jennifer somewhere between a teacher and a friend, which was not what Jennifer expected and thus troubled her. Introverted and shy, Jennifer did not know how to handle her relationship with the students. She chose to wear clothes of dark colors and not to crack jokes with the students over some issues to appear more mature and maintain a proper distance with them.

### Jennifer’s Cognitions About Her Professional Development in the Second Stage

In 1997, after achieving good scores in University organized examinations, Jennifer was chosen to attend a 3-year master program for English teachers and sent to East China Normal University (Shanghai). Her in-service degree advancement was financially sponsored by the university. She regarded this experience as a “giant leap” in professional development.

*During my study in East China Normal University, I was shown to the door to the academic palace. In the first year, I took specialized courses in English and was amazed that the knowledge learned in that 1 year was even more than that in my four university years.*

Jennifer recalled how her teachers, masters of EFL teaching and research, helped her grow and develop as an EFL teacher. For example, Professor Liu Naiyin, the compiler of many textbooks for extensive reading courses for English majors, organized readings of English plays and novels, which opened Jennifer’s eyes to English linguistics and literature. The platform of East China Normal University, rated by Jennifer as “much better than that of interior universities,” organized practical activities and exchange visits to other universities in Shanghai.

With profound knowledge and improved EFL teaching skills learned in her masters program, Jennifer became more confident in her EFL teaching. She started to teach English majors (freshmen and sophomores) Integrated English in a 2 year cycle. With improved oral expression, Jennifer deemed that she had established the image of an excellent EFL teacher; praises in various forms from her students further increased her confidence. In this second stage, Jennifer’s cognitions about EFL teaching, her role(s) in EFL teaching, and her relationship with students developed. Jennifer defined this stage as her “comfort zone.”

#### Jennifer’s Cognitions About English as a Foreign Language Teaching in the Second Stage

As previously mentioned, Jennifer’s masters research supplied her with a more profound basis of the English language in terms of teaching philosophy and the perspective from which English literature was viewed. Jennifer had a new cognition of issues in EFL teaching such as textbooks, teaching focus, grading homework, and the teacher’s attitude toward students asking questions in class. The textbook for the course of Integrated English was *Contemporary College English* (Books One to Four) compiled by Professor Yang Limin from Beijing Foreign Studies University, published and distributed by Foreign Language Teaching and Research Press. Jennifer sang highly of the textbook:

*The textbook Contemporary College English is very good, especially the exercises. If you carefully check the exercises with the text to locate the original sentences accordingly and finish the exercises concerning phrases and sentence patterns attentively, you will master the text for a basic view.*

Jennifer’s cognition regarding teaching focus improved. In the past, she focused on implementing lessons, and trying to explain the literal meaning of texts. Comparatively, now, Jennifer recognized the importance of higher level components – sentences: She started to relate textual comprehension to structural analysis of sentences. Jennifer explained that the analysis of long and difficult sentences facilitated textual appreciation:

*I had an enlightenment about how sentence analysis can help facilitate text appreciation. I never tried to analyze the long and difficult sentences in the texts before; now I am interested in teaching the students how long sentences are formed inside and how to analyze them grammatically.*

Jennifer developed a deep understanding of grading students’ homework (especially compositions). Based on her perceived improvement in grading students’ written homework, Jennifer noted that grading homework was essential for students’ academic development and therefore was an important part of an EFL teacher’s responsibility. To elaborate on her idea, she contrasted teacher’s grading with peer grading:

*Students’ homework, especially compositions, can only achieve its expected function when graded by the teacher. Peer grading is of little use in this field for two reasons. First, as some students barely have any knowledge about English writing, how can they do a rational grading of their peers’ work? Second, driven by a high mark for their own compositions, students tend to give their peers too high marks to be suitable.*

The fourth issue in Jennifer’s cognition about EFL teaching was a teacher’s response to students’ questions for which they could not provide ready answers. She classified teachers’ responses into three main types: tending to treat questions as a challenge to their authority easily perceiving offense, promising to answer them in the next class while never keeping the promise, and attentively promising and keeping the promises. Jennifer lent her favor to the third type of teachers by saying the following words:

*The teacher should take a reasonable attitude toward students’ questions. It is understandable that the teacher has not prepared profoundly enough for classroom teaching or even make mistakes; it is unforgivable if the teacher blames the students for raising questions or does not keep his or her promise of offering an explanation next time.*

#### Jennifer’s Cognitions About Her Role(s) in English as a Foreign Language Teaching in the Second Stage

With expanded cognitions about EFL teaching and accumulated teaching experience, Jennifer’s role in the classroom evolved in competency as an EFL teacher and an organizer of classroom activities. In the interviews, Jennifer appeared in high spirits when asked about her classroom role(s) in the second stage, which indicated her satisfaction in terms of EFL instructional practice. With deeper textual understanding and improved teaching competence, Jennifer started to pay attention to the students, wondering how to activate the class.

*My attention used to be on implementing lessons; now I start to think about the students’ responses and learning effect in the classroom. To liven up the class atmosphere and engage students in learning more deeply, I started to organize some activities for them.*

Driven by this cognition, Jennifer organized various classroom activities for her students. For example, the author of *Thinking as a Hobby* (Text A in Unit One of Book Four) displayed contempt toward mass wisdom by grading 9/10 of the population as third grade thinkers – the lowest grade of thinking, full of ignorance and prejudice. The author’s ideas were diametrically opposed to Chinese people’s shared attitude toward collective wisdom. To focus students’ attention on the difference and call for their understanding, a group discussion was organized in class. Students, working in groups of four or five, hotly debated this issue. Themes in the text were explored and accepted based more on the students’ understanding than the teacher’s inculcation.

#### Jennifer’s Cognitions About Her Relationship With Students in the Second Stage

In developing her EFL teaching competence, Jennifer began to impose higher requirements on the students and obsessively focus on students’ academic performance as measured by exam grades. Gradually, she developed a dislike toward under-achieving students regardless of their efforts in group discussions and homework; as a result, her relationship with under-achievers remained in tension.

*Students’ bounden duty is to study hard and achieve good grades in exams. With all the conditions provided by parents and teachers, if students cannot achieve well, they must be lazy or distracted.*

Jennifer exhibited a sense of responsibility, an indication of development relative to the first phase where she was concerned with implementing lessons and improvement in herself in terms of English proficiency and teaching skills. In this stage, she developed an awareness of students’ academic performance through testing. Driven by this sense of responsibility, Jennifer invested more time in tutoring students’ learning and monitoring their morning reading and evening autonomous learning classes, which achieved positive results in improving students’ scores in exams.

The tension between Jennifer and her under-achieving students was alleviated when those students became juniors and didn’t have to attend Jennifer’s classes any more as the *Integrated English* course was only for freshmen and sophomores. Jennifer found those students who entered the Students’ Union demonstrated great consideration, meticulousness and outstanding communicative ability. She started to realize that these qualities were as equally important as academic performance for the students’ career options and development after graduation because they might not continue in academics as their livelihood in the future.

### Jennifer’s Cognitions About Her Professional Development in the Third Stage

Jennifer was promoted to be the Associate Director of the Department of Foreign Languages in 2010 and Department Director in 2017 when she shouldered the administrative job and EFL teaching post at the same time. The administrative job required her to contact numerous people related to her position including personnel at all university and college levels, all the teachers and students in the Department. This experience in administration and EFL teaching broadened her vision as an EFL teacher and affected her cognitions.

#### Jennifer’s Cognitions About English as a Foreign Language Teaching in the Third Stage

Promoted to the Department Director position, Jennifer had a wider purview of English language teaching and education. In terms of content, Jennifer deemed the integration of morals education into EFL classroom teaching as important and necessary; while in terms of teaching methods, Jennifer saw increased value in the use of modern information technology in the EFL classroom.

Morals education which aims to ingrain a nation’s unique traditions, culture, values, and moralities into students, contributes prominently to students both as individuals and as community members. According to Jennifer, morals education should be an important component which needs to be integrated into EFL teaching.

*EFL teaching is more than mere imparting English knowledge and language skills; it should also take the responsibility of educating and cultivating students to be distinguished world citizens. I often integrate moral education into classroom teaching when explaining texts.*

Jennifer shared one example of her integrating morals education into classroom instructions. The text entitled *The Man in the Water* related a true story about how an anonymous passenger repeatedly passed his lifeline and flotation ring to others in the icy river when a helicopter rescue team came following a plane crash. Beyond explaining the literal meaning and language points of the text, Jennifer talked a lot about selflessness, heroism, and the relationship between man and nature. Furthermore, she asked students to share their thoughts on these topics to the class and engaged them in discussions, inspiring students to relate the heroic deed to real life situations. Through exploration, the students acquired a deeper understanding about sacrifice, righteousness, the position to take between man and nature, thereby accomplishing the goal of morals education.

Modern information technology has brought about fundamental changes to EFL education, making teaching more student-focused by involving them in what is happening instead of a traditional lecture format. As educational technology becomes more interactive, increased student engagement with learning materials occurs. With online learning platforms, students learn by doing, researching, and receiving immediate feedback, activating their passion for further exploration. Group work, involving discussions, debates, or cooperative learning becomes more accessible with Internet tools fostering teamwork.

*Compared against the chalk and blackboard in the past and the computer and curtain screen installed in the classroom later, the modern information technology devices are more advanced and multifunctional, rendering our EFL classroom teaching into learning fun, breaking the limit of time and space. I have never thought of this before, and it’s so amazing; after the trials during COVID-19, I love to integrate it into my EFL classroom instruction now.*

Compelled by COVID-19, teachers were required to use modern educational technology for online teaching, which facilitated the integration of modern technology into all levels of education. Now literate and equipped, Jennifer enjoyed integrating modern educational technology into her EFL instruction effectively making use of online educational platforms offered by the university.

#### Jennifer’s Cognitions About Her Role(s) in English as a Foreign Language Teaching in the Third Stage

As the Department Director, Jennifer faced not only her students, but also students in the Department as well, which enriched her cognition of the students as a whole thereby elevating her role to a comprehensive educator concerned for the students’ overall development and employability upon graduation.

*I find myself more concerned about the students’ inner feelings and started to look at things from their perspectives. I am now in a position to help them not only learn for final exams but also develop qualities necessary for future development.*

Jennifer intentionally spent time in training students for employability. For example, she told the students to be aware of the audience when presenting in the class instead of mechanically reciting prepared content by elaborating on public speaking as a significant means of verbal communication.

*You cannot avoid verbal communication with others in your future job. Even if you are an engineer burying yourself in the desk work, you will have to convince your clients of your design. What would happen if you read your PowerPoint slides without even having a look at your client?*

To foster students’ overall development, Jennifer put more weight on the students’ internalization of knowledge and increasing the scope of this knowledge. She often cited Chinese classic works of literature and philosophy to enlighten her students during her classroom instruction. Once Jennifer explained the word *justice* to the students. She first told the story of how Emperor Justinian of Byzantine Empire helped compile the *Justinian Code* which laid the foundation of western legal code, and then explored the three basic principles of justice with the students: being honest, hurting nobody, and giving everyone his or her due.

*Be honest with yourself as well as others; otherwise, you need to lie about lying. Hurt nobody and do not revenge when you are stronger, because that will lead to mutual destruction haunting you to the end of your life. Give everyone his or her due, i.e., what he or she deserves from previous behaviors, because that leaves no follow-up troubles.*

Another example is Jennifer’s explanation of a verse from *The Analects of Confucius*: Isn’t it a pleasure to learn and apply from time to time what one has learned (the original Chinese version is 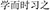, 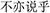)? She recounted that the Chinese character 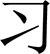 (xi) here did not mean *to review or to go over* (what one has learned); instead, the character meant to *put* (what one has learned) *into practice*, which accounted for the reason why it was translated into *apply*.

*By saying this, Confucius was urging his disciples to combine knowledge and practice together.*

She then further explored the implicit meaning of this character with her students and encouraged them to apply what they had learned into everyday practice and make it a habit for learning to be able to have great achievements. Jennifer also cited Confucius’s own account of his progress and attainments to give her students advice on things to do in different age bands: “At 15, I had my mind bent on learning. At 30, I had planted my feet firm upon the ground. At 40, I no longer suffered from perplexities. At 50, I knew the biddings of Heaven. At 60, I heard them with docile ear. At seventy, I could follow my hearts desires, for what I desired no longer overstepped the boundaries of right (the original Chinese version is 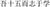; 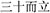; 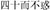; 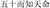; 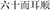; 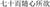, 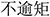).”

By relating language points to the construction of students’ inner values and world outlook, Jennifer was performing her role of an advisor or a supervisor who guided the students to make the right choices and remaining on the right track no matter which stage of life they were in. She often advised students to read books of genuine wisdom and philosophy and think profoundly for the cultivation of inner peace and emotional and mental detachment from material benefits.

#### Jennifer’s Cognitions About Her Relationship With Students in the Third Stage

In this stage, Jennifer increasingly stayed with students and observed them more. She grasped the temporal features of the students over time. According to Jennifer, her students in recent years were different from those in the past; they established their generations identity as being heavily reliant on digital devices and lacking in real-life communication.

*They are like geeks of digital devices to me. They rely heavily on their smartphones, pads, or laptops, etc. They are well adapted to online communication; they feel free in the cyberspace. I was aware of this in online lessons during COVID-19. Students were very lively there, responding to my questions actively, sending funny Emoji or memes, and cracking jokes with me and their classmates.*

The students’ heavy reliance on digital devices is partly due to the development of IT technology and subsequent development of applications which enabled people to do work and daily chores with their devices, especially apps for learning and playing. The negative outcome is that they were more comfortable with online communication; they lacked interest and practice in real life communications. The resultant vitality in online lessons and inactive response for offline lessons was therefore unavoidable. To Jennifer’s disappointment, the hot discussions and the lively atmosphere seldom happened in offline classes. The students remained silent as they did before COVID-19, offering few responses when Jennifer asked group questions.

*They ducked their heads, avoiding eye contact. That made me feel irritated yet sympathetic. I couldn’t help wondering if they were the same group of students in online lessons.*

Jennifer explained that the students might feel nervous to communicate in the face-to-face way since they were used to online communication where psychologically they felt much safer because of the physical distance, since their faces were hidden behind the screen; when they sat together with each other with the teacher standing in front of them they were prisoners of their nerves and anxieties.

## Discussion

**FIGURE 1:**
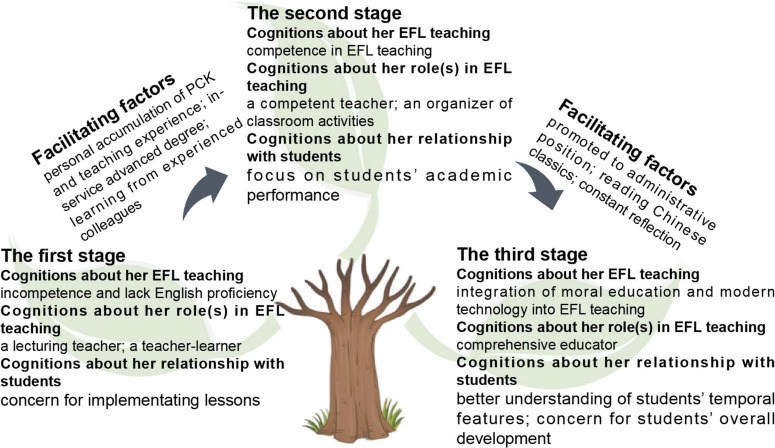
The trajectory of Jennifer’s professional development as an EFL teacher.

The present study explored an EFL teacher’s professional development trajectory over the past 28 years with regard to her cognitions about her EFL teaching, her role(s) in EFL teaching, and her relationship with students. The constant comparison of data about Jennifer’s experiences elicited from her written overview and interviews has revealed her evolution from a novice teacher to an experienced teacher, and finally to a teacher leader. Those factors that facilitated her professional development were also investigated. Framed by the research questions, the discussion was conducted in view of the theory and reviewed literature. Many changes occurred during Jennifer’s development from a novice EFL teacher to an experienced one, and then to a teacher leader. Figure 1 displays the trajectory of Jennifer’s professional development in the three stages and the facilitating factors.

As Figure 1 shows, from her origins as a novice teacher to the stage of an experienced teacher, mediated by her accumulation of PCK knowledge and teaching experience, the knowledge obtained from an in-service advanced degree, and learning from experienced colleagues, Jennifer’s cognitions about her EFL teaching, her roles in EFL teaching, and her relationship with students changed from an anxious and worried teacher-learner concerned with lesson implementation to a competent EFL teacher and classroom activity organizer focusing on students’ academic performance. From one stage as an experienced teacher to another as a teacher leader, facilitated by her promotion to an administrative position and reading Chinese classics, Jennifer’s cognitions upgraded again into a comprehensive English educator who integrated morals education and modern technology into EFL teaching and concern for students’ overall development.

The present study revealed that Jennifer’s cognitions about her professional development are dynamic, not static or fixed; she plays an active role in the process instead of being a passive receiver, which echoes Beijaard et al. (2004). One of the facilitating factors was Jennifer’s in-service advanced degree, which is in line with Darling-Hammond’s (2010b) study and Dillon’s (2011) research that universities could offer financial assistance for teachers’ in-service training and learning for higher degrees to facilitate the teacher development. This finding is consistent with Sun and Zhang’s (2019) exploration that teachers’ own understanding of their teaching behaviors helps shape teacher cognition.

The research also revealed that Jennifer’s cognitions about her professional development over the past 28 years as an in-service EFL teacher were contextually situated and mediated by her professional coursework (for example, her advanced degree) and other contextual factors such as the university, external demands, her personal life, and her classroom teaching practice (for example, organizing classroom activities). It is a case in point that adds evidence to model of language teacher cognition Borg’s (2015). This finding is also in alignment with many studies on cognition-practice relationship of language teachers (for example, Borg, 2015; Farrell and Ives, 2015; Ozturk and Gurbuz, 2017; Li, 2020; Zhan and Jiang, 2021).

Reflection was a key factor that mediated her cognitions about her professional development and brought cognition and practice closer together in the third stage. This finding supports Postholm; Postholm’s (2008; 2012) results showing reflection is the key to teachers’ learning and professional development. Jennifer’s cognitions were dynamically shaped in the chronological process of professional development by the facilitating factors which in turn shaped her professional development. This is in line with what Berliner (1994), Richards and Lockhart (1996); Kang and Cheng (2014), Ozturk and Gurbuz (2017); Li (2020), Li and Liu (2021); Zhan and Jiang (2021), and Liu et al. (2022), etc., have found in their studies.

## Conclusion

This qualitative study was designed to explore an EFL teacher’s cognitions about her professional development over the past 28 years. The findings have theoretical implications for research on language teacher cognition. It has modestly expanded the knowledge of an EFL teacher’s cognitions by profoundly investigating the longitudinal changes through the three stages of professional development. This study has also provided new empirical proof for language teacher cognition about not only what teachers think but also about how an EFL teacher’s cognitions shapes her professional development and how professional development shapes her cognitions in turn. This was reinforced especially during the pandemic epidemic period because the findings related to facilitating factors in each stage are new to field of language teacher cognition research. English language teacher educators and EFL teachers in the Chinese tertiary context or other similar contexts around the globe may find pedagogical implications in the findings of the present research. It has contributed to a better understanding that language teacher cognition is contextually specific and meaningful and needs to be further explored in other dimensions. English language teachers can learn through participation in various forms or co-operation with colleagues, planned or unplanned, formal or informal. Facing students from different generations with various learning needs, language teachers need to constantly upgrading their cognitions remaining flexible and be prepared to learn new skills to take on rapidly evolving challenges.

Despite its significance, the present study has an evident shortcoming: The number of participants was limited to one and Jennifer’s case from a novice EFL teacher to an experienced EFL teacher, and finally to a teacher leader does not apply to most EFL teachers since not all EFL teachers have the opportunity to be promoted to director of a department; their professional development may stop at being experienced teachers or evolve into teacher-researchers in an academic dimension. Future research might include more EFL teachers, to explore their development from novice to the teacher-researcher stage for a more holistic and generalizable approach to explore the facilitating factors of their professional development. In addition, data elicitation from other sources such as classroom observations may provide other investigative avenues.

## Data Availability Statement

The raw data supporting the conclusions of this article will be made available by the authors, without undue reservation.

## Ethics Statement

Ethical review and approval was not required for the study on human participants in accordance with the local legislation and institutional requirements. The patient(s)/participant(s) provided their written informed consent to participate in this study. Written informed consent was obtained from the participants for the publication of any potentially identifiable images or data included in this article.

## Author Contributions

LG conceptualized the study, collected the data, analyzed them, wrote the first draft, and contributed to the revising of the first draft and subsequent revisions. JY took a part in the research design and provided all the data. Both authors contributed to the article and approved the submitted versions.

## Conflict of Interest

The authors declare that the research was conducted in the absence of any commercial or financial relationships that could be construed as a potential conflict of interest.

## Publisher’s Note

All claims expressed in this article are solely those of the authors and do not necessarily represent those of their affiliated organizations, or those of the publisher, the editors and the reviewers. Any product that may be evaluated in this article, or claim that may be made by its manufacturer, is not guaranteed or endorsed by the publisher.
